# Third-line therapy for chronic myeloid leukemia: current status and future directions

**DOI:** 10.1186/s13045-021-01055-9

**Published:** 2021-03-18

**Authors:** Jorge Cortes, Fabian Lang

**Affiliations:** 1 Georgia Cancer Center at Augusta University, 1410 Laney Walker Rd., CN2222, Augusta, GA 30912 USA; 2 Department of Medicine, Hematology and Oncology, Goethe University Hospital, Building 33, 3rd floor, Room 246, Theodor-Stern-Kai 7, 60590 Frankfurt a. Main, Germany

**Keywords:** Chronic myeloid leukemia, Third line, Tyrosine kinase inhibitors, Emerging therapies

## Abstract

Chronic myeloid leukemia (CML) is driven by the BCR-ABL1 fusion protein, formed by a translocation between chromosomes 9 and 22 that creates the Philadelphia chromosome. The BCR-ABL1 fusion protein is an optimal target for tyrosine kinase inhibitors (TKIs) that aim for the adenosine triphosphate (ATP) binding site of ABL1. While these drugs have greatly improved the prognosis for CML, many patients ultimately fail treatment, some requiring multiple lines of TKI therapy. Mutations can occur in the ATP binding site of ABL1, causing resistance by preventing the binding of many of these drugs and leaving patients with limited treatment options. The approved TKIs are also associated with adverse effects that may lead to treatment discontinuation in some patients. Efficacy decreases with each progressive line of therapy; data suggest little clinical benefit of treatment with a third-line (3L), second-generation tyrosine kinase inhibitor (2GTKI) after failure of a first-generation TKI and a 2GTKI. Novel treatment options are needed for the patient population that requires treatment in the 3L setting and beyond. This review highlights the need for clear guidelines and new therapies for patients requiring 3L treatment and beyond.

## Introduction

Chronic myeloid leukemia (CML) is characterized by the presence of the Philadelphia (Ph) chromosome formed by a balanced translocation between chromosomes 9 and 22, leading to formation of a *BCR-ABL1* fusion gene [1]. The resultant constitutively active BCR-ABL1 fusion oncoprotein drives the pathogenesis of CML [2]. The advent of tyrosine kinase inhibitors (TKIs), which target the adenosine triphosphate (ATP) binding site of ABL1, has transformed CML into a chronic disease; many patients achieve a life expectancy close to that of the general population [3]. Treatment and response recommendations currently focus on first-line (1L) and second-line (2L) therapy; 1L treatment is usually a first-generation (imatinib) or second-generation (2G; nilotinib, dasatinib, or bosutinib) TKI, as outlined in the National Comprehensive Cancer Network (NCCN) guidelines and European LeukemiaNet (ELN) recommendations [4, 5]. As treatments have advanced over time, treatment goals have evolved from improving survival, preventing progression, and reducing treatment-related toxicities to treatment-free remission (TFR); however, some patients fail to meet these goals with the use of existing approved therapies [4–9]. Patients who achieve a sustained deep molecular response (DMR) on TKI therapy may be eligible for TFR [8, 9]. For patients who are not eligible for TFR, DMR is still an important treatment goal, as DMR has been associated in some studies with improved overall survival (OS) [10]. However, a significant proportion of patients fail to reach sustained DMR. Among patients with CML in chronic phase (CML-CP), over 50% of patients treated with imatinib eventually develop resistance or intolerance [11, 12]. For 2GTKIs, when used as frontline therapy, approximately 30–40% of patients need to change therapy by 5 years [13–15]. By 5 years, only ≈ 30% of patients treated with imatinib and 30–55% treated with 2GTKIs achieved a 4.5-log molecular response (MR^4.5^, *BCR-ABL1*^IS^ ≤ 0.0032%) [15–17].

While clear guidelines exist for 1L and 2L therapies, treatment beyond 2L is poorly established as few studies have prospectively addressed this scenario [4, 5]. Patients with treatment failure/resistance to 2L therapy have limited options and exhibit poor responses to additional treatment, with few achieving DMR [5, 18–23]. An unmet need exists for more efficacious third-line (3L) options for patients resistant or intolerant to TKIs.

## Unmet needs in the 3L+ setting

Many patients with CML are at risk of disease progression; sequential TKI use is associated with a decreased probability of response and worse OS. By 5 years, 30–50% of patients discontinue imatinib, with 5–7% discontinuing due to intolerance and 15–20% due to resistance [16, 17]. Patients with poorer molecular responses to imatinib are at a higher risk of progression and death [10]. Resistance rates are even higher during 2L treatment, with 60–70% of patients failing to achieve a major molecular response (MMR) and 50–56% of patients failing to achieve a complete cytogenetic response (CCyR) with 2 years of follow-up (Table 1) [14, 24–26]. Patients with failure on 3L TKI therapy have higher rates of progression and death [11, 18, 19, 27].

**Table 1:** Response rates of 1L and 2L TKI therapies^a^

Study	Number of patients, n	Arms	Response	Response rate, n (%)	Patients remaining on study at data cutoff, n (%)
1L					
Hochhaus A, et al. *Leukemia.* 2016 [16]	283	Imatinib 400 mg QD	MMR by 5 y MR^4^ MR^4.5^	171 (60.4) 118 (41.7) 89 (31.4)	141 (49.8)^b^
	282	Nilotinib 300 mg BID	MMR MR^4^ MR^4.5^	217 (77.0) 185 (65.6) 151 (53.5)	169 (59.9)
	281	Nilotinib 400 mg BID	MMR MR^4^ MR^4.5^	217 (77.2) 177 (63.0) 147 (52.3)	174 (61.9)
Cortes J, et al. *J Clin Oncol.* 2016 [17]	260	Imatinib 400 mg QD	MMR by 5 y MR^4.5^	(64) (33)	162 (63)
	259	Dasatinib 100 mg QD	MMR MR^4.5^	(76) (42)	158 (61)
Cortes J, et al. *J Clin Oncol.* 2018 [14]	241^c^	Imatinib 400 mg QD	MMR at 2 y MR^4^	(50.7) (25.7)	
	246	Bosutinib 400 mg QD	MR^4.5^ MMR MR^4^ MR^4.5^	(10.8) (61.2) (32.8) (13.1)	
2L					
Kantarjian H, et al. *Blood.* 2011 [24]	321^d^	Nilotinib 400 mg BID	MCyR by 2 y MMR	(59)^e,f^ 82 (28)^g^	124 (39)
Shah N, et al. *Haematologica.* 2010 [25]^h^	167	Dasatinib mg 140 QD	MCyR by 2 y CCyR MMR^i^	105 (63) 84 (50) 55 (38)	
	167	Dasatinib 100 mg QD	MCyR CCyR MMR	106 (63) 83 (50) 57 (37)	
	168	Dasatinib 70 mg BID	MCyR CCyR MMR	103 (61) 90 (54) 56 (38)	
	168	Dasatinib 50 mg QD	MCyR CCyR MMR	103 (61) 84 (50) 59 (38)	
Gambacorti-Passerini C, et al. *Am J Hematol.* 2014 [131]	200	Bosutinib (imatinib resistant)	MCyR^j^ CCyR^j^ MMR^k^	108 (58) 85 (46) 45 (34)	92 (46)
	88	Bosutinib (imatinib intolerant)	MCyR^l^ CCyR^l^ MMR^m^	49 (61) 43 (54) 24 (35)	37 (42)

*1L* first line, *2L* second line, *BID* twice a day, *CCyR* complete cytogenetic response, *MCyR* major cytogenetic response, *MMR* major molecular response, *MR*^*4*^ 4.0-log molecular response (*BCR-ABL1*^IS^ ≤ 0.01%), *MR*^*4.5*^ 4.5-log molecular response (*BCR-ABL1*^IS^ ≤ 0.0032%), *QD* once a day, *TKI* tyrosine kinase inhibitor

^a^These are selected studies focusing on pivotal trials

^b^Patients remaining on core study treatment at 5 years

^c^268 patients were randomized to each arm; however, 54 failed screening; 3 patients randomly assigned to imatinib arm were not treated

^d^Patients were resistant or intolerant to imatinib

^e^45% if measured only by metaphase analysis and excluding fluorescence in situ hybridization analysis and also excluding MCyR responders who had an MCyR at baseline or who had a missing cytogenetic analysis at baseline

^f^56% for imatinib-resistant patients and 66% for imatinib-intolerant patients

^g^Molecular response was assessed in 294 of the 321 patients

^h^Patients remaining on study at data cutoff were not included in the publication

^i^600 patients were assessed for molecular response

^j^186 evaluable patients

^k^132 evaluable patients

^l^80 evaluable patients

^m^68 evaluable patients

Current TKIs have off-target activities due to their lack of specificity, which can lead to long-term safety issues and intolerance, and 2–24% of patients discontinue therapy because of adverse events (AEs) due to on- or off-target effects [11, 14, 16–19, 27–32]. Despite management of therapy-related AEs with dose reductions, transient treatment interruptions, supportive care, and concomitant medications, many patients treated with ≥ 2 TKIs are still at higher risk of experiencing TKI intolerance [33]. Cross-intolerance is uncommon in patients treated with TKIs, with the possible exception of myelosuppression, which is reported more frequently; however, patients may experience different AEs upon switching therapy [34–37]. As is the case with resistance, for patients with intolerance to 2L therapy, there are few remaining options with favorable benefit: risk profiles [38]. Because of limited effective options beyond 2L, patients may need to continue their 2L or 3L therapy despite experiencing AEs, frequently at doses not conducive to optimal response.

Sequential treatment with TKIs is frequently accompanied by the emergence of new mutations, resulting in limited sensitivity to the remaining TKIs [39]. The BCR-ABL1 T315I mutation confers resistance to all approved ATP-competitive TKIs, except ponatinib [4, 5, 7, 40]. In mutation analyses conducted in a series of studies of patients with imatinib failure with or without prior interferon-α, the frequency of T315I mutation was reported to be 10–27% among patients with a BCR-ABL1 mutation and 3–15% overall. In the 2L setting, the frequency of T315I mutation was reported in 9–53% of those with a BCR-ABL1 mutation and in 2–14% overall [41]. Options for patients with T315I mutations are limited to ponatinib, omacetaxine (only approved in USA), and allogeneic stem cell transplant (allo-SCT). These options have several potential limitations, such as safety concerns, limited efficacy, and adverse impact in quality of life. In addition, other BCR-ABL1 mutations (e.g., T315M and T315V [rare]) or compound mutations (e.g., Y253H/T315I or E255V/T315I) may confer resistance to ponatinib [42–44]. Because of the risk of arterio-occlusive events associated with ponatinib (and other existing TKIs), those with cardiovascular risk factors have even fewer treatment options [4, 5, 7, 38].

## Available 3L therapies

The standard-of-care beyond 2L therapy is not well defined by NCCN and ELN guidelines [4, 5, 7]. Upon resistance to and/or intolerance to 2L TKIs, any of the remaining TKIs may be used [4, 7], although there are limited data, frequently only anecdotal or case series for some of them. The choice of a 3L TKI may depend on a patient’s comorbidities, prior AEs, mutation profiles, drug interactions, and compliance issues [36, 45].

Ponatinib is a third-generation (3G) TKI approved for patients with CML resistant to ≥ 2 TKIs and for patients with T315I mutations [38, 46]. Per ELN 2020 recommendations, ponatinib is preferred over an alternative 2GTKI in patients without significant cardiovascular risk factors who are resistant to a 2GTKI without specific mutations [5].

Omacetaxine, a protein translation inhibitor, is available in the USA for patients who are resistant or intolerant to ≥ 2 TKIs and does not target the kinase domain of BCR-ABL1 [47, 48]. In a study of 76 highly pretreated patients with CML-CP, 18.4% experienced a major cytogenetic response (MCyR), with 7.9% obtaining a CCyR and 3.9% obtaining a partial cytogenetic response (PCyR) [49, 50]. In 35 patients with CML in the accelerated phase (AP), 14.3% obtained a major hematologic response with complete hematologic response (CHR) in 11.4% and no evidence of leukemia in 2.9% [49, 50]. The primary toxicity of omacetaxine is myelosuppression, which can be severe and prolonged.

Allo-SCT remains an important option for patients with CML-CP with failure after ≥ 2 TKIs [5, 45]. Allo-SCT may also be considered for patients with de novo CML in blast phase (BP), preferably after achieving some response with a TKI-based therapy, in patients with CML-AP who are not responding well to current therapy, in patients with progression to CML-AP/BC while receiving TKI therapy, and in those with resistance or intolerance to TKIs [4, 5]. It may also be used in patients with T315I mutations after an inadequate response to attempted ponatinib therapy [45]. Patients with CML-CP undergoing allo-SCT within the first year of diagnosis have a 5-year survival rate of ≈ 70%, while those receiving allo-SCT after this time have a 5-year survival rate of 60% [51]. The 3-year survival rate is 86% with busulfan plus cyclophosphamide prior to hematopoietic cell transplant, and ≈ 90% of these patients achieve molecular remission [51]. Potential complications with this treatment include graft-vs-host disease, and outcomes may be influenced by phase of disease, age, and the stem cells used [51].

## Therapeutic goals in 3L+ patients

ELN 2020 and NCCN have developed recommendations/guidelines for assessment of response in 1L and 2L treatment, lack guidance for 3L therapy and beyond, reflecting the lack of sufficient data in this setting. According to ELN 2020 recommendations, patients are considered to fail 2L therapy and recommended to switch to 3L therapy if *BCR-ABL1*^IS^ > 10% is confirmed within 1 to 3 months of therapy, *BCR-ABL1*^IS^ > 10% by 6 months, *BCR-ABL1*^IS^ > 1% by 12 months, or *BCR-ABL1*^IS^ > 1% at any time with resistance mutations or other high-risk chromosomal abnormalities in Ph + cells [5]. Per NCCN guidelines, patients are considered to fail 2L therapy and recommended to switch to 3L therapy if *BCR-ABL1*^IS^ > 10% at 6 and 12 months [4]. However, the clinical benefit of switching to 3L therapy for patients meeting these definitions has not been demonstrated.

The acceptable response to 3L + treatment remains undefined by ELN and NCCN. However, *BCR-ABL1*^IS^ > 1% or lack of a CCyR is considered an insufficient response for optimal survival, predicting a high risk of disease progression in these cases [4, 5]. Data thus far demonstrate that use of an alternative 2GTKI in patients who experience failure on multiple TKIs is not regularly associated with high rates of response and the responses achieved are not usually durable [4, 27, 52]. Resistance to therapy can be caused by both novel mutations in *BCR-ABL1* and non–BCR-ABL1–mediated mechanisms [39, 53, 54].

## Clinical trials with a 3L 2GTKI after failure of imatinib and a 2GTKI

Many reports, mostly case series, of 3L 2GTKIs following failure of imatinib and another 2GTKI have demonstrated poor long-term outcomes (summarized in Table 2).

**Table 2:** Responses seen in studies of 3L therapy

3L study	Efficacy
A retrospective study of patients receiving 2L dasatinib or nilotinib after imatinib [55]	Significant correlation between higher rates of CCyR and DMR and no treatment interruption with ≤ 2L 5-year OS was 83.0% in total and 94.5% in patients with CML-CP
A report of patients treated with 3 sequential TKIs [27]	Best response to 3L 2GTKI in 48 patients was MMR in 5 patients, CCyR in 3 patients, partial or minor CyR in 5 patients, and CHR in 6 patients
A report of patients treated with dasatinib or nilotinib after failing imatinib [57]	Rates of MCyR, CCyR, and MMR were 50.0%, 34.6%, and 19.2%, respectively
A study of patients receiving 3L nilotinib or dasatinib [58]	CHR, MCyR, CyR, CCyR, and MMR rates were 31.7%, 7.3%, 14.6%, 17.1%, and 15.9%, respectively, with 14.6% having no response Overall, 14% of patients died
A retrospective study of patients on 3L [52]	MCyR and CCyR were achieved in 15 of 45 and 11 of 52 patients, respectively Overall, 13 patients died
A single-center study of nilotinib or dasatinib in patients who failed 2 prior TKIs [59]	CHR, CCyR, and MMR were achieved in 89%, 13%, and 24%, respectively, of patients with CML-CP Of patients with CHR, 56% lost that response within a median of 23 months 5-year OS, PFS, and EFS were 86%, 54%, and 22%, respectively

*2GTKI* second-generation tyrosine kinase inhibitor, *2L* second line, *3L* third line, *CCyR* complete cytogenetic response, *CHR* complete hematologic response, *CML-CP* chronic myeloid leukemia in chronic phase, *CyR* cytogenetic response, *DMR* deep molecular response, *EFS* event-free survival, *MCyR* major cytogenetic response, *MMR* major molecular response, *OS* overall survival, *PFS* progression-free survival, *TKI* tyrosine kinase inhibitor

### *Bosi *et al*. *[55]

A retrospective study was conducted to evaluate patient characteristics and outcomes in a cohort of 90 patients with CML without access to new or investigational therapies who received 1L imatinib; those experiencing progression were treated with 2L or 3L dasatinib or nilotinib. Most patients had CML-CP (90%); 6.7% had CML-AP; and 3.3% had CML-BC. Thirty-five patients (38.8%) were relapsed, refractory, or intolerant to imatinib, and 13 (14.5%) needed ≥ 3 lines of therapy. A significant correlation was found between higher response rates (CCyR and DMR) and no treatment interruption and patients not needing > 2L therapy. Five-year OS in the total population was 83% and was 94.5% in patients with CML-CP (excluding deaths not related to CML). Five-year OS decreased to 82% and 77% in patients receiving 2L and 3L+ therapies, respectively.

### *Garg *et al*. *[27]

In this report, a total of 48 patients were treated with 3 sequential TKIs—34 of whom were treated with dasatinib after imatinib and nilotinib and 14 with nilotinib after imatinib and dasatinib. Before the start of 3L therapy, 25 patients were in CP. Best response to 3L 2GTKI was an MMR in 5 patients, a CCyR in 3 patients, a partial and minor CyR in 5 patients, a CHR in 6 patients, and no response in 6 patients, with a median failure-free survival of 20 months. Three patients with CML-CP who achieved a CCyR had responses lasting > 12 months.

### *Giles *et al*. *[56]

This analysis assessed the efficacy of nilotinib after failure of 1L imatinib and 2L dasatinib. Sixty patients with Ph + CML-CP/AP were enrolled to receive nilotinib 400 mg twice a day (BID). The median duration of follow-up was 12 months, with 3L nilotinib treatment ongoing at the time of the report in 22 patients. The most common reasons for discontinuation in patients with CML-CP included progression (11 patients) and AEs (4 patients). The median duration of nilotinib exposure was 11 months in patients with CML-CP. Of the 37 evaluable patients with CML-CP, 22 of 28 (79%) without a CHR at baseline achieved a CHR, 16 (43%) achieved an MCyR, and 9 (24%) achieved a CCyR. CHR was maintained until data cutoff, and the duration of MCyR ranged from 3.2 to 23 months. The 4 patients with baseline T315I mutations (including 2 with CML-CP) did not respond to nilotinib therapy.

### *Ibrahim *et al*. *[57]

In this cohort of 26 patients with CML-CP, 20 had been treated with dasatinib and 6 with nilotinib after failing imatinib in different phase II trials. Median follow-up after the start of 3L therapy was 21.5 months. During follow-up, 42.3% of patients failed 3L therapy and 34.6% died. MCyR, CCyR, and MMR rates were 50.0%, 34.6%, and 19.2%, respectively. Multivariate analyses showed that a CyR achieved on imatinib or 2L therapy was an independent predictor of a CCyR with 3L therapy, and achievement of a CyR with 2L therapy was the only independent predictor of an MCyR. Patients with a CCyR on 1 of the 2 previous therapies had a significantly higher probability of achieving a CCyR in the 3L setting. Achievement of a CCyR on 2L therapy and age < 64 years were independent predictors of OS. All patients with primary cytogenetic resistance to both 1L and 2L therapies failed to achieve a CCyR on 3L TKI therapy.

### *Russo Rossi *et al*. *[58]

This manuscript reports outcomes of 3L nilotinib or dasatinib therapy in patients with failure on 2 prior TKIs. A total of 82 patients received 3 sequential TKIs: 34 patients received 3L dasatinib, of whom 30 (88.2%) were in CP; 48 patients received 3L nilotinib, of whom 38 (79%) were in CP. Responses to 3L TKI therapy included 13 (15.9%) patients with an MMR, 14 (17.1%) with a CCyR, 12 (14.6%) with a PCyR, 6 (7.3%) with an MCyR, 26 (31.7%) with only a CHR, and 12 (14.6%) with no response. Response rates were transient; 30–50% of patients did not achieve a CCyR within 12 months. In patients receiving 3L dasatinib, 41.2% discontinued due to toxicity and 26% experienced transformation. In patients receiving 3L nilotinib, 50% discontinued due to toxicity and 21% experienced transformation. As patients went through more TKIs, an increased frequency of mutations was observed in patients. Overall, 14% of patients died, and the onset of T315I mutation was associated with an increased risk of death.

### *Lomaia *et al*. *[52]

This retrospective study assessed outcomes in 53 patients on 3L therapy; 18 were treated with nilotinib, 33 with dasatinib, and 5 with bosutinib. Forty-eight patients discontinued previous TKI therapy because of resistance, with 42 patients experiencing resistance to both prior TKIs. MCyR and CCyR were achieved in 15 of 45 and 11 of 52 patients with median durations of 9.3 and 4.5 months, respectively. Intolerance was the main reason for treatment discontinuation (5 patients). Progression on or after therapy occurred in 8 patients, with a median time to progression of 14.7 months. Two-year OS was 67%. All patients with an MCyR were alive and maintained CP; however, 13 patients died on study.

### *Ribeiro *et al*. *[59]

This single-center study evaluated nilotinib or dasatinib in patients with failure after 2 prior TKIs. The objective was to assess hematologic, cytogenetic, and molecular responses and progression-free survival (PFS), event-free survival (EFS), and OS in patients treated with a third TKI. Of the 25 patients evaluated, 9 were treated with 3L dasatinib and 16 with 3L nilotinib. Eighteen patients had CML-CP, of whom 89% achieved a CHR; 13% achieved a CCyR, and 24% achieved an MMR. Fifty-six percent of patients with CML-CP who had a CHR lost that response within a median of 23 months. Five-year OS, PFS, and EFS were 86%, 54%, and 22%, respectively, in patients with CML-CP, and 66%, 66%, and 0% in patients with CML-AP. All patients with CML-BC died during this study. Responses obtained using a 3L TKI were generally not sustained; however, the authors suggested that this therapy might be useful as a temporizing measure until a donor becomes available for allo-SCT.

## Clinical trials with omacetaxine in 3L+

Omacetaxine is a semisynthetic formulation of homoharringtonine that induces apoptosis in BCR-ABL1–bearing cells by down-regulating MCL1 and also by inhibiting protein synthesis through binding to ribosomes at their A-cleft [47]. Omacetaxine is approved in the USA for patients with CML-CP resistant or intolerant to ≥ 2 TKIs, including patients with T315I mutation after TKI failure [49]. Data were pooled from 2 open-label, single-arm, phase II studies [60, 61]. Patients received induction therapy (omacetaxine 1.25 mg/m^2^ BID subcutaneously for up to 14 consecutive days every 28 days until hematologic response) followed by maintenance therapy (omacetaxine 1.25 mg/m^2^ BID for up to 7 days per 28-day cycle, for up to 24 months or until progression or toxicity) [50]. A total of 81 patients with CML-CP enrolled in this study [50]. Of 76 evaluable patients, 53 (70%), 14 (18%), and 7 (9%), respectively, achieved a CHR, an MCyR, and a CCyR. Twenty-two patients had T315I mutations at baseline, of whom 18 (82%), 5 (23%), and 3 (14%), respectively, achieved a CHR, an MCyR, and a CCyR. Of 40 patients who received 2 prior TKIs, 31 (78%), 10 (25%), and 5 (13%), respectively, achieved a CHR, an MCyR, and a CCyR; of 36 patients who received 3 prior TKIs, 22 (61%), 4 (11%), and 2 (6%), respectively, achieved a CHR, an MCyR, and a CCyR. The median PFS values for the evaluable population and for patients who received > 3 cycles of therapy were 9.6 and 9.9 months, respectively. The median OS values for the evaluable population and for those who received > 3 cycles were 40.3 and 49.3 months, respectively [50].

The most common nonhematologic AEs of any grade were diarrhea (43%), nausea (38%), fatigue (30%), infections (26%), pyrexia (22%), headache (22%), asthenia (22%), and arthralgia (20%). Grade 3/4 thrombocytopenia, neutropenia, and anemia occurred in 67%, 48%, and 40% of patients with CML-CP, respectively. Serious AEs occurred in 46 patients (57%) with CML-CP. The most common any-grade hematologic serious AEs occurring in ≥ 5% of patients were bone marrow failure (11%), thrombocytopenia (11%), and febrile neutropenia (7%); no nonhematologic serious AEs occurred in ≥ 5% of patients with CML-CP. Two deaths occurred on study or within the first 30 days of follow-up (due to disease progression and multiorgan failure [*n* = 1 each]); none were related to study drug [50].

Long-term administration of omacetaxine was feasible and safe; dose adjustments were frequently required to manage myelosuppression. However, MCyR and CCyR rates were modest (< 25%) in all patient cohorts. Patients with > 3 cycles of omacetaxine treatment showed a trend toward longer PFS and OS compared with the overall population, but only a small number of patients with CML-CP demonstrated durable responses. Overall PFS was < 10 months, and overall OS was < 4 years [50]. Because of its modest clinical activity, omacetaxine is mostly used in patients who have used or cannot use any of the available TKIs and are not eligible for allo-SCT.

## Prospective clinical trials of TKIs in 3L

### Phase I/II trial to determine efficacy and safety of 3L+ bosutinib in patients with CML-CP resistant or intolerant to imatinib plus dasatinib and/or nilotinib [18, 62]

Adult patients with Ph + CML-CP who received imatinib followed by dasatinib and/or nilotinib were enrolled in this prospective study. The cohort of patients analyzed was either imatinib resistant (≥ 600 mg/day) or imatinib intolerant (any dose) and had ≥ 1 of the following: resistance to dasatinib (≥ 100 mg/day), intolerance to any dose of dasatinib, resistance to nilotinib (800 mg/day), intolerance to any dose of nilotinib, or resistance/intolerance to dasatinib and nilotinib. Dose escalation to bosutinib 600 mg/day was allowed in patients with no CHR by week 8 or no CCyR by week 12, except in patients with grade ≥ 3 treatment-emergent AEs (TEAEs).

There were 41 (14%) patients still on bosutinib after ≥ 9 years of treatment. At ≥ 8 years of follow-up, the median duration of treatment was 26 months overall and median duration of follow-up was 54 months. Most patients (90/119; 76%) discontinued therapy by 4 years, with a further 21 patients discontinuing therapy since then. Main reasons for discontinuation included AEs (28; 24%), progressive disease (24; 20%), or lack of efficacy (22; 18%). Half the patients received dose reductions because of AEs. The 4-year cumulative confirmed CHR, MCyR, and CCyR rates were 74%, 40%, and 32%, respectively. The Kaplan–Meier (KM)–estimated probabilities of maintaining confirmed CHR and MCyR at 4 years were 63% and 69%, respectively. The MCyR rate differed among the 3 main subsets of imatinib resistant/intolerant patients: additional dasatinib intolerance, 87%; additional nilotinib resistance, 78%; and additional dasatinib resistance, 43%. At 4 years, cumulative incidence of on-treatment progressive disease or death was 24% overall, with a total of 26 (22%) on-study deaths. The KM-estimated 4-year OS was 78%, and 9-year OS was 74%. A minimal CyR with prior dasatinib and/or nilotinib predicted survival, and a lower Ph + ratio at baseline (≤ 35%) predicted achievement of an MCyR or a CCyR in this study. Most responses were observed within the first year of treatment and discontinuation rates were high, with 68% of discontinuations (due to AEs) occurring during year 1.

TEAEs were reported in all 119 patients, with grade 3/4 TEAEs being reported in 81 (68.1%) patients. The most common TEAEs included diarrhea (83%), nausea (48%), vomiting (38%), and thrombocytopenia (39%). Overall, 33 (28%) patients discontinued treatment because of AEs. Cross-intolerance to bosutinib was reported in 20% of imatinib-intolerant patients and in 24% of dasatinib-intolerant patients.

### Ponatinib efficacy and safety in Ph + leukemia: final 5-year results of the phase II PACE trial [19]

Ponatinib is a 3G ATP-competitive TKI with activity against T315I and all other tested BCR-ABL mutations. Adult patients with CML or Ph + acute lymphoblastic leukemia (ALL) resistant or intolerant to dasatinib or nilotinib, or patients with CML-CP with T315I mutation regardless of prior TKI therapy, were enrolled in the pivotal PACE trial. Ponatinib was administered at a starting dose of 45 mg once a day (QD). Dose adjustments were allowed to manage AEs; however, because of concerns about arterio-occlusive events (AOEs), dose reductions to 15 and 30 mg QD were recommended in October 2013 (2 years after enrollment ended in October 2011) for all patients with CML-CP with or without an MCyR, respectively.

In the final 5-year follow-up report of PACE, among the cohort of 270 patients with CML-CP, 64 (24%) of whom had the T315I mutation, the median duration of treatment was 32.1 months, with a median follow-up of 56.8 months. Fifty-seven (21%) patients discontinued because of AEs, 29 (11%) discontinued because of disease progression, and 15 (6%) discontinued because of lack of efficacy.

The CyR was evaluable in 267 patients: 159 (60%) achieved an MCyR at any time, of whom 144 (54%) achieved a CCyR; 108 (40%) achieved an MMR; and 64 (24%) achieved MR^4.5^. Median times to MCyR, CCyR, and MMR among those who achieved the response were 2.8, 2.9, and 5.5 months, respectively. Of those who achieved an MCyR at 12 months and an MMR at any time, 82% and 59% of patients, respectively, maintained responses at 5 years. The KM-estimated PFS and OS at 5 years were 53% and 73%, respectively.

The most common TEAEs (≥ 40%) were rash (47%), abdominal pain (46%), thrombocytopenia (46%), headache (43%), dry skin (42%), and constipation (41%). The most common grade 3/4 TEAEs (≥ 10%) were thrombocytopenia (35%), neutropenia (17%), hypertension (14%), increased lipase (13%), abdominal pain (10%), and anemia (10%). AOEs were reported in 84 (31%) patients and were serious in 69 (26%) patients: cardiovascular, cerebrovascular, and peripheral vascular events occurred in 42 (16%), 35 (13%), and 38 (14%) patients, with serious events in 33 (12%), 28 (10%), and 31 (11%) patients, respectively. Thirty-five (13%) patients had dose adjustments as a result of AOEs. Five patients had grade 5 AOEs; among patients with CML-CP, these included acute myocardial infarction (*n* = 1), cerebrovascular accident (*n* = 1), and hemorrhagic cerebral infarction (*n* = 1); patients with Ph + ALL experienced mesenteric arterial occlusion (*n* = 1) and peripheral ischemia (*n* = 1).

Overall, 56% of patients with CML-CP achieved the primary endpoint of MCyR by 12 months. The cumulative incidence of AOEs continued to increase over time, with events occurring overwhelmingly among patients with additional risk factors for such events. Ponatinib is thus a valuable option for patients who have received prior therapy, but safety considerations have limited its use. An ongoing study (NCT02467270, OPTIC study) is assessing the optimal dose schedule for ponatinib to strike a balance between efficacy and safety [63]. In this study, patients were randomized to receive either ponatinib at 45 mg daily (cohort A), 30 mg daily (cohort B), or 15 mg daily (cohort C). Upon achieving *BCR-ABL1*^IS^ ≤ 1%, the patients receiving 45 mg or 30 mg daily reduced their doses to 15 mg daily. Preliminary analyses show that 39%, 27%, and 26% of patients in cohorts A, B, and C, respectively, achieved *BCR-ABL1*^IS^ ≤ 1% at 12 months. AOEs occurred in 5%, 4%, and 1% of patients in cohorts A, B, and C, respectively, with serious AOEs reported in 2%, 3%, and 0%, respectively. Discontinuations due to TEAEs occurred in 18%, 15%, and 14% of patients in cohorts A, B, and C, respectively, with 4 (1.4%) deaths on study [63]. Ponatinib was recently approved for use in patients who have failed ≥ 2 TKIs, with dose reductions down to 15 mg daily upon achievement of response [38]. Still, other 3L + options are needed in patients who fail to achieve responses on ponatinib or who may not be optimal candidates because of the risk of AOEs (Table 3).

**Table 3:** Efficacy and safety results from prospective clinical trials of TKIs in the 3L

Trial	Efficacy	Safety
A phase I/II trial of 3L + bosutinib in patients with CML-CP resistant/intolerant to imatinib + dasatinib and/or nilotinib (Study 200) [18]	At 4 years: 74% cumulative cCHR 63% probability of maintaining cCHR 40% cumulative MCyR 69% probability of maintaining MCyR 24% cumulative incidence of on-treatment CML-AP/BC or death and 22% on-study deaths 78% KM-estimated OS	TEAEs reported in 100% of patients and grade 3/4 TEAEs reported in 68.1% of patients Most common TEAEs were diarrhea (83%), nausea (48%), vomiting (38%), and thrombocytopenia (39%)
Ponatinib efficacy and safety in Ph + leukemia [19, 46]	Overall, in patients with CML-CP [19]: 60% achieved MCyR at any time, of whom 54% achieved CCyR 40% achieved MMR 24% achieved MR^4.5^ 3% of patients transformed to CML-AP/BC KM-estimated PFS and OS at 5 years was 53% and 73%, respectively In a study of patients with CML-CP and resistance/intolerance to nilotinib or dasatinib or who had a T315I mutation [46]: 51% of patients with intolerance/resistance and 70% with T315I mutation achieved MCyR, with 40% and 66% achieving CCyR, respectively MMR was achieved in 27% of patients with resistance/intolerance to nilotinib or dasatinib and 56% of patients with a T315I mutation 12% of patients discontinue use due to AEs	In patients with CML-CP [19]: Most common TEAEs (≥ 40%) were rash (47%), abdominal pain (46%), thrombocytopenia (46%), headache (43%), dry skin (42%), and constipation (41%) Most common grade 3/4 TEAEs (≥ 10%) were thrombocytopenia (35%), neutropenia (17%), hypertension (14%), increased lipase (13%), abdominal pain (10%), and anemia (10%) 31% of patients had AOEs In a study of patients with CML-CP and resistance/intolerance to nilotinib or dasatinib or who had a T315I mutation [46]: Common AEs were thrombocytopenia (37%), rash (34%), dry skin (32%), and abdominal pain (22%) Serious arterial thrombotic events occurred in 9% of patients, with 3% considered to be treatment related 12% of patients discontinued use because of AEs

*3L* third line, *AE* adverse event, *AOE* arterio-occlusive event, *cCHR* cumulative confirmed complete hematologic response, *CCyR* complete cytogenetic response, *CML-AP* chronic myeloid leukemia in acute phase, *CML-BC* chronic myeloid leukemia in blast crisis, *CML-CP* chronic myeloid leukemia in chronic phase, *KM* Kaplan–Meier, *MCyR* major cytogenetic response, *MMR* major molecular response, *MR*^*4.5*^ 4.5-log molecular response (*BCR-ABL1*^IS^ ≤ 0.0032%), *OS* overall survival, *Ph+* Philadelphia chromosome positive, *PFS* progression-free survival, *TEAE* treatment-emergent adverse event, *TKI* tyrosine kinase inhibitor

## Overview of new BCR-ABL1–targeted therapies in development

New CML therapies are in development, with particular focus on 3L therapy and/or patients with a T315I mutation—settings for which treatment options remain limited and suboptimal. HQP1351 (olverembatinib) is a 3G BCR-ABL1 TKI with in vitro activity against T315I and other mutants, as well as nonmutated BCR-ABL. It has shown a manageable safety profile and significant and lasting efficacy in a phase I study in patients with CML who are resistant to current TKI therapies, particularly among those with T315I mutations [64–67]. Unlike other TKIs, HQP1351 does not form a hydrogen bond with the hydroxyl group of the BCR-ABL T315 residue, allowing it to bind in the presence of T315I mutations [66]. HQP1351 was orally administered (1–60 mg) every other day. The median duration of follow-up was 12.8 months. Among evaluable patients with CML-CP, 52 of 55 (94.5%) without a CHR at baseline achieved a CHR; 56 of 81 (81%) without a CCyR at baseline achieved an MCyR and 49 (60.5%) achieved a CCyR; and 32 of 86 (37.2%) without an MMR at baseline achieved an MMR. More patients with CML-CP harboring the T315I mutation achieved a CHR, an MCyR, a CCyR, and an MMR than those without the mutation [64]. Thrombocytopenia was the most common hematologic TEAE reported in patients, with any-grade and grade 3/4 thrombocytopenia reported in 75.2% and 49.5% of patients, respectively [64]. Preliminary results from a two-part phase II HQP1351 trial in heavily pretreated patients with CML-CP (study CC201) and CML-AP (study CC202) harboring the T315I mutation were recently published [68]. Patients in both studies were treated with HQP1351 40 mg once every other day for 28 consecutive days per cycle over 24 months. For study CC201, 41 patients enrolled in the trial and 92.7% completed at least 6 cycles of therapy. The 3- and 6-month PFS was 100% and 96.7% across the median duration of follow-up of 7.9 months. Among 31 evaluable patients without CHR at baseline, 30 (96.8%) achieved CHR. Among 41 evaluable patients without CCyR at baseline, 31 (75.6%) achieved MCyR (the primary objective of the study), including 27 (65.9%) and 4 (9.85) who achieved CCyR and PCyR, respectively. Of 41 evaluable patients, 20 (48.8%) achieved MMR. Frequent grade ≥ 3 treatment-related AEs were thrombocytopenia (48.8%), anemia (24.4%), neutropenia (19.5%), and leukopenia (12.2%). Frequent nonhematologic treatment-related all-grade AEs were skin pigmentation (53.7%) and elevated creatine kinase (48.8%), alanine aminotransferase (31.7%), and aspartate aminotransferase (26.8%) [68]. For study CC202, 23 patients enrolled in the trial and 78.3% completed at least 6 cycles of therapy. Across the median duration of follow-up of 8.2 months, the 3- and 6-month PFS was 100% and 95.5%. At baseline, 23 patients did not have a major hematologic response; 18 (78.3%) of patients achieved this response on study (the primary endpoint of this study). Of the 23 evaluable patients without MCyR at baseline, 14 (60.9%) achieved CHR, 12 (52.2%) achieved MCyR, and 6 (26.1%) achieved MMR. Common grade ≥ 3 treatment-related AEs were thrombocytopenia (52.2%), anemia (39.1%), leukopenia (30.4%), and neutropenia (21.7%). The most commonly reported nonhematologic treatment-related all-grade AEs were skin pigmentation (69.6%), hypocalcemia (52.2%), proteinuria (52.2%), hypertriglyceridemia (47.8%), hyperphosphatemia (43.5%), arthralgia (34.8%), and fatigue (26.1%) [68].

PF-114 is another orally available ATP-competitive TKI with efficacy at nanomolar concentrations against both wild-type and mutated BCR-ABL1, including the T315I mutation [69–71]. It is structurally similar to ponatinib but modified to avoid inhibition of vascular endothelial growth factor receptor in an attempt to minimize cardiovascular toxicity. A phase I/II dose-finding study in patients with Ph + CML-CP or CML-AP resistant to ≥ 2 TKIs and in patients harboring the T315I mutation enrolled 51 patients who received daily doses ranging from 50 to 750 mg [69–72]. At a follow-up of ≥ 6 months, therapy was ongoing in 17 patients. In the optimal safety and efficacy dose cohort (300 mg QD), 6 of 11 patients achieved an MCyR and 4 patients achieved an MMR. Of 12 patients with T315I mutations, 3 and 4 patients, respectively, achieved a CHR and an MCyR. Drug-related grade 3 skin toxicity, mostly in the form of psoriasiform lesions, was reported in 11 patients receiving ≥ 400 mg [70].

Vodobatinib (K0706) is another orally bioavailable BCR-ABL1 TKI designed using a structure-guided drug-design platform with significant activity in vitro against most BCR-ABL mutations, but not T315I [73]. Vodobatinib showed an acceptable safety profile in a phase I study in patients with CML who experienced treatment failure with ≥ 3 TKIs and/or patients with comorbidities that restrict the use of certain TKIs (nilotinib, dasatinib, and ponatinib) [74, 75]. At the time of data cutoff, 35 patients received doses ranging from 12 to 240 mg—27 of whom had CML-CP. Seven and 4 patients with CML-CP, respectively, achieved and maintained a CCyR; 5 achieved an MMR; and 2 achieved MR^4.5^. Of the 12 of 27 responders, 11 remained on treatment with a durable MCyR for 6.9 months; 1 had progression at 9.5 months. Mild to moderate gastrointestinal AEs were reported in 18.5% of patients. Two patients enrolled had T315I mutations; these patients experienced disease progression in cycle 1 of treatment, leading to a protocol amendment to exclude patients with T315I mutations from this study [74]. In a recently published exploratory analysis, efficacy, and safety of vodobatinib was assessed in ponatinib-pretreated and -naive patients with CML-CP, with the goal of determining MTD or RP2D. Patients received escalating doses of vodobatinib (12 to 240 mg once daily) in 28-day cycles. Sixteen and 15 patients, respectively, enrolled in the ponatinib-pretreated and -naive cohorts. The median duration of treatment was 17.3 months and 14.8 months, respectively. Efficacy was comparable between the 2 cohorts with 50% ponatinib-pretreated and 67% naive patients having CCyR. Most common treatment-emergent all-grade AEs were myalgia (33%), back pain (27%), thrombocytopenia (27%), and nasopharyngitis (20%); 3 CVEs unrelated to study treatment were reported in 2 patients (1 in each cohort); 1 ponatinib-pretreated patient died on study due to disease progression and 2 naive patients due to pneumonia and intracranial hemorrhage (*n* = 1 each) [76].

**Fig. 1:**
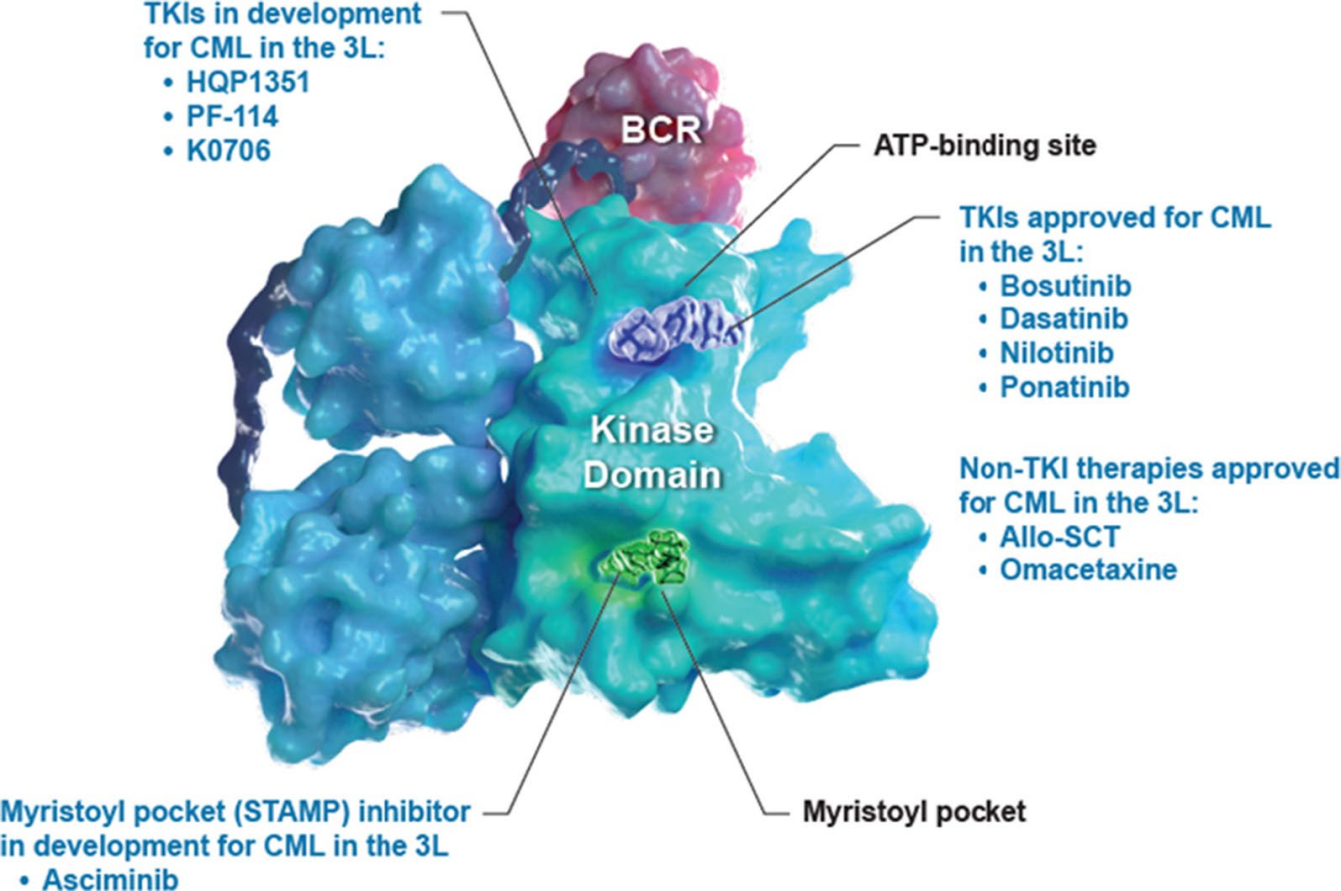
Therapies in development vs approved therapies for CML in the 3L + setting. *3L* third line, *allo-SCT* allogeneic stem cell transplant, *ATP* adenosine triphosphate, *CML* chronic myeloid leukemia, *STAMP* Specifically Targeting the ABL Myristoyl Pocket, *TKI* tyrosine kinase

Asciminib is a first-in-class BCR-ABL1 inhibitor Specifically Targeting the ABL Myristoyl Pocket (STAMP inhibitor) [77, 78]. Unlike TKIs that target the ATP binding site, asciminib has a unique mechanism of action, binding to the myristoyl pocket of ABL1 and inhibiting the fusion protein in a non–ATP-competitive manner (Fig. 1). Normally, a myristoyl group will bind the myristate pocket of ABL1, inducing an inactive state and regulating kinase activity; however, this mechanism of autoregulation is lost upon fusion with BCR. Because of its different mechanism of action compared with currently available TKIs, it has a nonoverlapping mutation-driven resistance profile with approved TKIs and maintains activity against BCR-ABL1 with ATP-site resistance mutations, including the T315I mutation [79, 80]. Preclinical data has shown that asciminib specifically inhibits the growth of BCR-ABL1–driven cancer cells, unlike TKIs, which are nonspecific inhibitors of BCR-ABL1. Because of its unique mechanism of action and specificity, asciminib monotherapy was predicted to provide improved efficacy compared with ATP-competitive TKIs in patients with resistance/intolerance to multiple prior TKIs, with a decreased risk of off-target effects [79–81]. The efficacy and safety of asciminib is being assessed in phase I, II, and III clinical trials [82–86].

Because asciminib targets the myristoyl pocket of ABL1, it can bind in combination with ATP-competitive TKIs [79, 80]. Preclinical studies showed that asciminib in combination with nilotinib (an ATP-competitive TKI) led to complete tumor regression in mice; when used separately, it led to the emergence of resistance mutations [80]. The complementary resistance profiles of asciminib and imatinib, dasatinib, or nilotinib had an additive effect in vitro, similar to that seen with asciminib in combination with ponatinib [42, 80]. The combination of asciminib and ponatinib at clinically relevant concentrations was effective against compound mutations, including T315I-inclusive compound mutations, and reduced ponatinib-associated toxicities (Table 4) [42]. Combinations of asciminib and ATP-competitive TKIs are being investigated in various clinical studies [86–89].

**Table 4:** Ongoing clinical trials for BCR-ABL1–targeted therapies for CML in 3L + setting

Drug/trial	Trial number/phase	Goals	Primary endpoint(s)
HQP1351 (BCR-ABL1 Inhibitor) [132, 133]	NCT04126681/phase II	To evaluate the efficacy of HQP1351 in patients with CML-CP who are resistant and/or intolerant to 1G and 2GTKIs	EFS
	NCT03883087/phase II	To evaluate the efficacy of HQP1351 in patients with CML-CP and a T315I mutation	MCyR
PF-114 (BCR-ABL1 Inhibitor) [72]	NCT02885766/phase I/II	To evaluate tolerability, safety, pharmacokinetics, and preliminary efficacy of PF-114 in patients with Ph + CML who are resistant to 2GTKIs or have the T315I mutation	DLTs MTD
K0706 (BCR-ABL1 Inhibitor) [75]	NCT02629692/phase I/II	To determine safety, tolerability, pharmacokinetics, and activity of K0706 in patients with CML or Ph + ALL	MTD TEAEs MCyR or partial cytogenetic response (CML-CP) CHR (CML-AP/BC)
Asciminib (STAMP inhibitor) [82, 86]	NCT03106779/phase III	To compare the efficacy of asciminib with that of bosutinib in patients with CML-CP in the 3L + setting	MMR at 24 weeks
	NCT02081378/phase I	A dose-finding study of asciminib alone or in combination with nilotinib, imatinib, or dasatinib in patients with CML and Ph + ALL who are relapsed/refractory to or are intolerant of TKIs	MTD and/or RDE DLTs

*1G* first generation, *2GTKI* second-generation tyrosine kinase inhibitor, *3L* third line, *ALL* acute lymphoid leukemia, *CHR* complete hematologic response, *CML-AP* chronic myeloid leukemia in acute phase, *CML-BC* chronic myeloid leukemia in blast crisis, *CML-CP* chronic myeloid leukemia in chronic phase, *DLT* dose-limiting toxicity, *EFS* event-free survival, *MCyR* major cytogenetic response, *MMR* major molecular response, *MTD* maximum tolerated dose, *Ph+* Philadelphia chromosome positive, *RDE* recommended dose for expansion, *STAMP* Specifically Targeting the ABL Myristoyl Pocket, *TEAE* treatment-emergent adverse event, *TKI* tyrosine kinase inhibitor

## Asciminib in the 3L+ setting

A phase I, dose-finding study (NCT02081378) of asciminib alone or in combination with imatinib, nilotinib, or dasatinib enrolled patients aged ≥ 18 years with Ph + CML-CP or CML-AP who relapsed or were refractory to ≥ 2 different TKIs or had unacceptable AEs from TKIs. Patients with the T315I mutation were enrolled if they had received ≥ 1 TKI and if no other treatment was available. Notably, in the expansion cohorts, the dose used for patients with a T315I mutation was considerably higher than that for other patients, based on preclinical data suggesting that higher concentrations are required. The doses used were within the range found safe in the phase I portion of the study. The study is ongoing, but results from the monotherapy cohort have been published [81], and preliminary results from the combination cohorts have also been reported [90, 91].

In the monotherapy cohort, 141 patients with CML-CP and 9 patients with CML-AP were enrolled and received asciminib QD or BID (10–200 mg). A maximum tolerated dose was not identified, but the recommended dose for expansion was determined to be 40 mg BID for patients without and 200 mg BID for those with the T315I mutation. Efficacy results were analyzed by T315I status and CML phase, and safety results were analyzed for the combined cohort of 150 patients [81]. The 5 most common all-grade TEAEs were fatigue (29.3%), headache (28.0%), increased lipase levels (26.7%), arthralgia (24.0%), and nausea (24.0%) [81].

In patients without the T315I mutation, 92% of patients without a CHR at baseline achieved a CHR; 60% of patients achieved an MCyR; 54% of patients without a CCyR at baseline achieved a CCyR; and 24% and 36% of evaluable patients achieved an MMR by 6 and 12 months, respectively, including 23% and 40% of patients, respectively, who were resistant or intolerant to ponatinib (Table 5) [81].

**Table 5:** Efficacy and safety results from the asciminib monotherapy cohorts of the phase I dose-finding study

Cohort	Efficacy	Safety
Cohort without T315I mutations [81]	37% (37/99) and 48% (44/91) of all evaluable patients achieved or maintained MMR by 6 and 12 months, respectively 77% (85/110) and 70% (77/110) of all evaluable patients achieved or maintained MCyR and CCyR, respectively	Study drug–related AEs of any grade were reported in 100% (150/150) of patients, and grade 3/4 AEs were reported in 60% (90/150) of patients The most common AEs of any grade were fatigue (29.3%), headache (28.0%), and increased lipase (26.7%)
Cohort with T315I mutations [81]	21% (4/19) and 24% (4/17) of all evaluable patients achieved MMR by 6 and 12 months, respectively 55% (11/20) and 41% (9/22) of all evaluable patients achieved MCyR and CCyR, respectively	
Cohort with baseline BCR-ABL1^IS^ ≤ 1% [77]	87.5% of patients remained on therapy at data cutoff 75% (18/24) of these patients were in MMR at data cutoff MR^4^ and MR^4.5^ were achieved by > 40% of evaluable patients who were not in MR^4^ or MR^4.5^ at baseline 12.5% discontinued due to AEs	Grade 3/4 AEs (in > 10% of patients), regardless of study drug, were increased lipase (27.1%) and hypertension (12.5%)

*AE* adverse event, *CCyR* complete cytogenetic response, *MCyR* major cytogenetic response, *MMR* major molecular response, *MR*^*4*^ 4.0-log molecular response (*BCR-ABL1*^IS^ ≤ 0.01%), *MR*^*4.5*^ 4.5-log molecular response (*BCR-ABL1*^IS^ ≤ 0.0032%)

In patients with the T315I mutation, 88% of patients without a CHR at baseline achieved a CHR; 55% of patients without an MCyR at baseline achieved an MCyR; 41% of patients without a CCyR at baseline achieved a CCyR; and 21% and 24% of evaluable patients achieved an MMR by 6 and 12 months, respectively, including in 14% and 17%, respectively, of patients who were resistant or intolerant to ponatinib (Table 5) [81]. Although MMR responses are higher among patients who had not previously received ponatinib (57.1% by 24 weeks), patients who had previously received ponatinib still had a 28.6% MMR rate by 24 weeks [92].

In a subanalysis of the ongoing phase I study, the safety and efficacy of asciminib monotherapy was evaluated in a subset of patients enrolled in the monotherapy cohort with baseline *BCR-ABL1*^IS^ ≤ 1%—a population considered to be primarily intolerant of TKIs. Asciminib monotherapy was well tolerated, with only 6.2% of patients discontinuing treatment because of AEs; at data cutoff, the median duration of drug exposure was 161 weeks. Asciminib was effective in this population as well, with 75%, 42%, and 43% of patients achieving an MMR, MR^4^, and MR^4.5^ overall by data cutoff of August 30, 2019, respectively—all of whom did not have those responses at baseline (Table 5). These results set the stage for an ongoing study exploring the use of asciminib in patients who do not achieve optimal outcomes with TKI therapies and miss therapeutic milestones [77].

The most frequent all-grade AEs (> 25% of patients), regardless of study drug relationship, were fatigue (43.8%), increased lipase (39.6%), headache (35.4%), increased amylase (29.2%), arthralgia (29.2%), diarrhea (29.2%), and abdominal pain (27.1%) [77]. The most frequent grade 3/4 AEs (> 5% of patients), regardless of study drug, were increased lipase (27.1%) and abdominal pain (12.5%) [77].

An ongoing phase III, multicenter, randomized study (ASCEMBL) is investigating the efficacy and safety of asciminib 40 mg BID vs bosutinib 500 mg QD in patients with CML-CP previously treated with ≥ 2 TKIs, with failure of or intolerance to the most recent TKI (NCT03106779) [82]. The goal of this study is to compare the efficacy of asciminib with that of bosutinib in the 3L + setting, with the rate of MMR at 24 weeks as the primary endpoint [82]. The primary efficacy and safety results from ASCEMBL (per May 25, 2020, data cutoff) have been reported: 233 patients with CML-CP were randomized in 2:1 ratio to asciminib 40 mg twice daily (*n* = 157) or bosutinib 500 mg once daily (*n* = 76), with a median duration of follow-up of 14.9 months from randomization to cutoff [93].

The study met its primary objective, with an MMR rate of 25.5% with asciminib and 13.2% with bosutinib at 24 weeks, and the treatment difference between the 2 treatment arms, after adjusting for the baseline stratification factor (MCyR status), was 12.2% (95% CI, 2.19–22.3: 2-sided *P* = 0.029). A homogenous and consistent superior treatment effect was observed with asciminib across most major demographic and prognostic subgroups, including in patients who received ≥ 3 prior TKIS, in those who discontinued their prior TKI due to treatment failure, and regardless of baseline MCyR status [93].

Asciminib had a better safety profile than bosutinib. Grade ≥ 3 AEs regardless of study drug relationship occurred in 50.6% of patients on asciminib compared to 60.5% on bosutinib. The most common grade ≥ 3 AEs occurring in > 10% of patients were thrombocytopenia (17.3%) and neutropenia (14.7%) with asciminib and neutropenia (11.8%), diarrhea (10.5%), and increased alanine aminotransferase (14.5%) with bosutinib. Two fatal events occurred on-treatment in the asciminib arm due to ischemic stroke and arterial embolism (*n* = 1 each) and 1 in the bosutinib arm due to septic shock [93].

## Novel therapies with non-BCR-ABL1 targets in CML

Key goals in the development of novel therapeutics include addressing non–BCR-ABL1–mediated CML leukemia stem cell (LSC) resistance and inhibiting other molecular pathways upregulated by or co-existing with active BCR-ABL1 signaling via the combination of TKIs with other agents [94–98]. CML LSCs are not eliminated by TKIs as they are not dependent on the kinase activity of BCR-ABL1 for their survival [99–101]. They have thus been suggested to play an important role in drug resistance and persistence, and to interact with the bone marrow microenvironment to evade drugs and host control mechanisms. These cells are thought to be one of the main causes for relapse, nonresponse, and resistance to TKI therapy and of relapse after treatment discontinuation as most TKIs in vitro are unable to eradicate these cells. Some of the novel therapies discussed here have not been effective, and/or clinical development of these therapies has been paused; others have shown preliminary efficacy in patients with CML, including some combinations of TKIs with other agents.

### TKIs in combination with other drugs

Currently approved TKIs combined with various anticancer agents (interferon-ɑ, chemotherapeutic agents, immunomodulators) may provide an additive or synergistic effect. Hence, several ongoing clinical trials are investigating the efficacy of approved TKIs with other agents [102]. Interferon-ɑ directly inhibits the proliferation of CML progenitor cells; the pegylated form is being studied in combination with bosutinib [103].

Expression of the immune checkpoint protein programmed cell death 1 ligand 1 (PD-L1) has been observed in patients with CML, particularly in patients classified as high risk by Sokal score [104]. Accordingly, expression of programmed cell death 1 protein (PD-1), the tandem immune checkpoint receptor for PD-L1, is also higher in the T cells of patients with CML [104]. These data suggest that targeting the PD-1/PD-L1 pathway may be an effective strategy for eliminating CML cells. Several clinical trials investigating immune checkpoint inhibitor therapy in combination with existing TKI therapies are ongoing. In a phase Ib trial investigating the PD-1 inhibitor nivolumab (1 or 3 mg/kg every 2 weeks) in combination with dasatinib, none of the 31 patients enrolled experienced a dose-limiting toxicity (NCT02011945, Study Results) [105]. Among patients with CML-CP with prior dasatinib exposure, 2 of 8 in the 1-mg/kg group achieved an MMR at 36 months, and 5 of 11 in the 3-mg/kg group achieved an MMR at 36 months [105]. In a separate phase I/II trial, several 2GTKIs in combination with the PD-L1 inhibitor avelumab are being investigated in patients with CML-CP [106].

Thiazolidinediones are peroxisome proliferator-activated receptor *γ* agonists that downregulate proteins overexpressed in LSCs. Results from a preclinical study showed that the peroxisome proliferator-activated receptor *γ* agonist pioglitazone sensitized CML cells to imatinib [102, 107]. An ongoing phase I/II study is investigating the combination of pioglitazone with imatinib [106].

A subset of CML cells (Ph+ CD34+) aberrantly express dipeptidylpeptidase IV—a protease that deregulates interactions between LSCs and the hematopoietic niche. Dipeptidylpeptidase IV inhibitors or gliptins can restore normal interactions between LSCs and the niche [102, 108]. Vildagliptin is being investigated in a phase I/II study in combination with nilotinib as a pretreatment in patients attempting TFR [109]. Other agents in clinical development or with preclinical activity in CML have been considered for the treatment of patients with CML in various settings. Listed below are some such agents; this list is not meant to be comprehensive or all-inclusive, because other agents in early development may also hold promise in various CML settings.

### JAK/STAT inhibitors

JAK activation leads to increased STAT phosphorylation, nuclear translocation, and transcriptional activity; activation of this pathway is observed in CML [110]. Preclinical studies have demonstrated that the JAK2 inhibitor ruxolitinib results in a reduction of quiescent CML LSCs [110]. In a phase I trial of ruxolitinib in combination with nilotinib in patients with CML-CP, a reduction in phosphorylated-STAT3 was observed after treatment, and 10 (40%) patients had undetectable *BCR-ABL1* transcripts [111]. The safety and efficacy of adding ruxolitinib to established therapy with bosutinib, nilotinib, or dasatinib is currently being studied in a phase II trial (NCT03654768) [112].

### Wnt/β-catenin inhibitors

Wnt/β-catenin pathway inhibitors may be effective for CML, because β-catenin—the canonical Wnt pathway’s central effector—is required for the development and maintenance of LSCs [113]. Preclinical studies have shown that combination of the Wnt/β-catenin inhibitor PRI-724 with nilotinib reduced the viability of quiescent CML cells in vitro and extended the survival of mice transplanted with TKI-resistant CML cells [113]. A phase I study was completed, but further clinical development is not ongoing [114].

### Liposome-incorporated antisense oligodeoxynucleotide

BP1001 is a liposome-incorporated antisense oligodeoxynucleotide that stops expression of growth factor receptor–bound protein 2 (Grb2)—a signal transducer. Grb2 mediates the activation of the oncogenic tyrosine kinases MAPK1 and MAPK3. As a single agent, BP1001 induced responses in a handful of patients with CML with resistance to multiple prior therapies [115]. BP1001 demonstrated efficacy and safety in combination with low-dose cytarabine in a phase I trial that enrolled patients with Ph + CML-CP, -AP, or -BC and other hematologic malignancies. BP1001 was administered intravenously, twice weekly for 28 days at a starting dose of 5 mg/m^2^ with dose escalations ranging from 10 to 90 mg/m^2^. Of 7 patients who received BP1001 plus cytarabine, 2 had complete remission, 1 had complete remission with incomplete hematologic recovery, and 2 had stable disease with no dose-limiting toxicities. The most common grade 3/4 AEs included cardiopulmonary disorders (64%) and fever (including neutropenic fever) and infections (44%) [115].

### TGF-β-FOXO-BCL-6

The TGF-β-FOXO-BCL-6 pathway is involved in the maintenance of LSCs; preclinical data show that treatment of mice with the TGF-β inhibitor LY364947 reduced LSC clonogenic activity in vitro [116].

### RAS inhibitors

Addition of a farnesyl moiety to RAS is a key post-translational step toward RAS activation, subsequently activating ERK signaling, which is observed with BCR-ABL1 activation. Inhibiting farnesyl transferase blocks RAS and downstream signaling [117]. Three farnesyl transferase inhibitors have been tested in CML: 2 in phase I clinical trials (tipifarnib and lonafarnib) [118, 119] and 1 (BMS-214662) in preclinical settings [120]. With tipifarnib, hematologic responses were attained in 17 (68%) of 25 assessable patients; 9 patients (36%) also achieved a CyR [118]. Lonafarnib use resulted in 3 patients (33%) with CML-CP achieving CHR [119]. BMS-214662 potently induced apoptosis of both proliferating and quiescent CML stem/progenitor cells with < 1% recovery of long-term culture-initiating cells [120]. Despite these findings, clinical development in CML of this class has been paused.

### Mechanistic target of rapamycin inhibitors

Constitutively active mechanistic target of rapamycin signaling is observed in CML and results in excessive cell proliferation and may contribute to resistance to chemotherapy [121]. Rapamycin (sirolimus) and RAD001 (everolimus) have been evaluated in phase I/II clinical trials; however, the trial evaluating rapamycin (NCT00776373) has been terminated, and 2 trials evaluating RAD001 have been completed without further clinical development [98].

### Histone deacetylase inhibitors

Histone deacetylase inhibitors have been studied in CML: preclinical studies have found that panobinostat in combination with imatinib was able to kill CML progenitor cells that were resistant to imatinib alone and could prevent tumor formation when injected into immunodeficient mice [122]. Other histone deacetylase inhibitors (pacrinostat, vorinostat) have demonstrated antileukemic activity [98]. A phase II study evaluated panobinostat monotherapy in patients with CML-CP with resistance to ≥ 2 TKIs [123]. One of 29 (3%) patients had a CHR, and 0 patients had an MCyR. Histone deacetylase inhibitors have questionable efficacy in monotherapy and may be best paired with TKIs.

### Hypomethylating agents

Hypomethylating agents are currently being investigated for use in CML in combination with TKIs, because hypermethylation of key genes—including BCR and ABL—has been reported in CML [124]. A study of decitabine in combination with dasatinib reported a major hematologic response, an MCyR, and an MMR in 48%, 44%, and 33%, respectively, of patients with CML-CP receiving the combination [125]. Another study assessed azacytidine in combination with TKIs in patients with CML who had a CCyR and minimal residual disease. Only 3 patients enrolled; however, they were able to achieve sustainable MR^4.5^ after azacytidine was added to their treatment regimens [124].

### Aurora kinase pathway inhibitors

The aurora kinase family regulates cell division, and dysregulation of their activity generates chromosomal abnormalities driving DNA alterations responsible for cell transformation; aurora kinase inhibitors are considered potential anticancer treatments [96, 98]. A phase II study assessed MK-0457 (tozasertib) monotherapy in patients with CML-CP and a T315I mutation [126]. Two of 15 patients (13.3%) with CML-CP achieved an MCyR. However, the response was minimal and was achieved only at higher, less-tolerable doses [126]. A phase I study assessed PHA-739358 (danusertib) monotherapy in patients with CML-AP/BC or Ph + ALL resistant or intolerant to imatinib and/or a 2GTKI. Four of 29 (13.8%) patients, all of whom had the T315I mutation, exhibited a hematologic response [127].

### BCL-2 inhibitors

B cell lymphoma protein 2, BCL-2, is a regulator of apoptosis and a potential target for CML therapy. Venetoclax, a BCL-2 inhibitor, has been tested for use in CML in the preclinical setting and has demonstrated increased apoptosis [128, 129]. In a retrospective study of patients with Ph + ALL and CML-BP, 50% of patients receiving venetoclax in combination with a TKI had a response [130].

## Conclusion

The development of TKI therapy has greatly improved the prognosis of patients with CML, allowing a shift in treatment goals from increasing survival to improving quality of life and attempting TFR [3–6, 8, 9]. However, despite these advances, 30–50% of patients experience failure of frontline imatinib therapy after 5 years, and many even when treated with frontline 2GTKIs; resistance rates are even higher for patients on 2L therapy, with 63–72% failing to achieve MMR with 2 years of follow-up [10, 16, 17, 24–26, 36]. Treatment guidelines for patients failing 2L therapy are lacking; little data have shown clinical benefit to switching to a different 2GTKI in the 3L setting [4, 5]. Therapies in development focus largely on new BCR-ABL1 TKI options, mostly ATP-competitive agents with some attractive early results in clinical trials. One new class has emerged, represented by asciminib, a novel first-in-class STAMP inhibitor. Asciminib has shown promising early-phase data and may help address unmet medical needs in later lines of therapy, such as resistance and intolerance [77, 79, 80]. Other pathways are being investigated as potential targets for CML, including immune signaling [105, 106] and the JAK/STAT [110–112] and mTOR [98, 121] pathways. Development of effective therapies for patients who fail 2L TKI therapies is still a critical unmet need for CML.

## Data Availability

Not applicable.

## Abbreviations

1L: First line
2G: Second generation
2GTKI: Second-generation tyrosine kinase inhibitor
2L: Second line
3G: Third generation
3L: Third line
AE: Adverse event
ALL: Acute lymphoid leukemia
allo-SCT: Allogeneic stem cell transplant
AOE: Arterio-occlusive event
AP: Accelerated phase
ATP: Adenosine triphosphate
BID: Twice a day
BP: Blast phase
cCHR: Cumulative confirmed complete hematologic response
CCyR: Complete cytogenetic response
CHR: Complete hematologic response
CML: Chronic myeloid leukemia
CP: Chronic phase
CyR: Cytogenetic response
DLT: Dose-limiting toxicity
DMR: Deep molecular response
EFS: Event-free survival
ELN: European LeukemiaNet
KM: Kaplan–Meier
LSC: Leukemia stem cell
MCyR: Major cytogenetic response
MMR: Major molecular response
MR^4^: 4.0-Log molecular response (*BCR-ABL1*^IS^ ≤ 0.01%)
MR^4.5^: 4.5-Log molecular response (*BCR-ABL1*^IS^ ≤ 0.0032%)
MTD: Maximum tolerated dose
NCCN: National Comprehensive Cancer Network
OS: Overall survival
PCyR: Partial cytogenetic response
PD-1: Programmed cell death 1 protein
PD-L1: Programmed cell death 1 ligand 1
PFS: Progression-free survival
Ph+: Philadelphia chromosome positive
QD: Once a day
RDE: Recommended dose for expansion
RP2D: Recommended phase 2 dose
STAMP: Specifically Targeting the ABL Myristoyl Pocket
TEAE: Treatment-emergent adverse event
TFR: Treatment-free remission
TKI: Tyrosine kinase inhibitor
